# Relationship between Periodontitis-Related Antibody and Frequent Exacerbations in Chronic Obstructive Pulmonary Disease

**DOI:** 10.1371/journal.pone.0040570

**Published:** 2012-07-11

**Authors:** Tamaki Takahashi, Shigeo Muro, Naoya Tanabe, Kunihiko Terada, Hirofumi Kiyokawa, Susumu Sato, Yuma Hoshino, Emiko Ogawa, Kazuko Uno, Koji Naruishi, Shogo Takashiba, Michiaki Mishima

**Affiliations:** 1 Department of Respiratory Medicine, Kyoto University, Kyoto, Japan; 2 Health Administration Center, Shiga University of Medical Science, Shiga, Japan; 3 Louis Pasteur Center for Medical Research, Kyoto, Japan; 4 Division of Endodontology, Iwate Medical University, Department of Conservative Dentistry and Oral Rehabilitation, Iwate, Japan; 5 Department of Pathophysiology–Periodontal Science, Okayama University Graduate School of Medicine, Dentistry and Pharmaceutical Sciences, Okayama, Japan; University of Cape Town, South Africa

## Abstract

**Background:**

To identify patients with chronic obstructive pulmonary disease (COPD) who are susceptible to frequent exacerbations is important. Although periodontitis aggravated by poor oral hygiene might increase the risk of lower respiratory tract infection, the relationship between periodontitis and COPD exacerbations remains unknown. This prospective cohort study investigates the relationship between periodontitis-related antibody and exacerbation frequency over a one-year period.

**Methods:**

We assessed an IgG antibody titer against *Porphyromonas gingivalis,* which is a major pathogen of periodontitis, and then prospectively followed up 93 individuals over one year to detect exacerbations.

**Results:**

The numbers of exacerbations and the rate of individuals with frequent exacerbations (at least two per year) were significantly lower in patients with higher IgG titer than those with normal IgG titer (0.8 vs. 1.2 per year, *p*  = 0.045 and 14.3 vs. 38.6%, *p*  = 0.009, respectively). Multivariate logistic regression analysis showed that being normal-IgG titer for periodontitis-related antibody significantly increased the risk of frequent exacerbations (relative risk, 5.27, 95% confidence interval, 1.30–25.7; *p*  = 0.019) after adjusting for other possible confounders, such as a history of exacerbations in the past year, disease severity, COPD medication and smoking status.

**Conclusions:**

Normal-IgG titer for periodontitis-related antibody can be an independent predictor of frequent exacerbations. Measuring periodontitis-related antibody titers might be useful to identify patients with susceptibility to frequent exacerbations so that an aggressive prevention strategy can be designed.

## Introduction

Chronic obstructive pulmonary disease (COPD) is the fourth leading cause of death worldwide, and it is associated with an increasing economic cost and social burden [1]. The natural history of COPD is punctuated by exacerbations, which consist of acute episodes of worsening symptoms that might warrant changes in regular medications. These exacerbations negatively impact lung function, health-related quality of life, prognosis and socioeconomic burden [1]. Thus, exploring predictors of exacerbations and identifying patients with susceptibility to frequent exacerbations are important to design an efficient preventive strategy.

Factors associated with exacerbation include disease severity [1], a history of exacerbations [2], smoking [3], chronic inflammation [4], bacterial colonization [5] and gastro-esophageal reflux disease [6]. In addition, we have reported an association between an impaired swallowing reflex and bacterial colonization, systemic inflammation and an increased risk of exacerbations [7]. Since an impaired swallowing reflex might cause the aspiration of oral bacteria leading to lower respiratory tract infection, and poor oral hygiene itself is involved in the risk of aspiration pneumonia [8]–[10], we speculated that poor oral hygiene would increase the frequency of exacerbations.

Periodontitis is a common oral infectious disease that is associated with poor oral hygiene among the general population. It is characterized by inflammation of the periodontium induced by subgingival plaque bacteria such as anaerobic gram-negative rods [11], that can also be associated with COPD exacerbation [12]. Chronic marginal periodontitis is more prevalent among patients with severe COPD than in other equally severe respiratory diseases [13] and the prevalence of periodontitis increases together with COPD severity [14]. In addition, serum antibody to *Porphyromonas gingivalis* (*P. gingivalis),* which is a frequently isolated pathogen, can be involved in systemic diseases such as cardiovascular disease [15]–[17]. However, the relationship between periodontitis and COPD exacerbation remains unclear.

We postulated that periodontitis is associated with COPD exacerbations. This prospective cohort study investigated the impact of baseline antibody titers for periodontal antigen (an index of periodontitis) on COPD exacerbation frequency for over one year. We also investigated the relationship between elevated-IgG titer for periodontitis-related antibody and inflammatory cytokines.

## Methods

### Ethics Statement

The study was approved by the ethics committee of Kyoto University (approval No. E182), and written informed consent was obtained from all participants.

### Protocol and Study Participants

We recruited 109 patients with COPD from an outpatient clinic at Kyoto University Hospital, Japan, between September 2006 and August 2008 for this study. All patients provided written informed consent to participate. Blood and induced sputum samples were collected under stable conditions (as defined below) at entry for subsequent assay. We excluded 16 patients based on the following criteria: female, Brinkman index <10 pack-years, respiratory diseases other than COPD, daily intake of systemic corticosteroids and complicated with malignant diseases within 5 years. Thus, 93 patients were prospectively followed up for over one year to detect exacerbations.

### Exacerbation Criteria

Exacerbations and stable periods were prospectively identified using diary cards as in our previous study [6], [7]. We adopted a modified version of the East London cohort study criteria to define COPD exacerbations [6] based on an increase in any two “major” symptoms (dyspnea, sputum purulence and sputum quantity) or an increase in one “major” and one “minor” symptom (wheeze, sore throat, cough and nasal congestion/discharge) for at least two consecutive days [18]. Stable condition was defined as an exacerbation-free interval of >4 weeks [6].

### Clinical Examinations

Pulmonary function tests (Chestac-65V; Chest MI Corp.; Tokyo, Japan) were performed after inhaling short-acting bronchodilators (salbutamol and ipratropium) at entry into the study. Lung volumes and diffusion capacity were measured using helium dilation and the single-breath method, respectively. The British Medical Research Council dyspnea scale (MRC) and Charlson Commorbidities index were also assessed.

Venous blood at entry was collected on the entry day during the stable period and stored at –80°C. Serum levels of immunoglobulin G (IgG) against *P. gingivalis* were measured using an enzyme-linked immunosorbent assay (ELISA) based on validated method [15], [19], [20]. Since the FDC381 and Su63 strains of *P. gingivalis* are serologically different, we separately measured antibody titers of *Pg*FDC381 and *Pg*Su63. We defined the cut-off point as the mean +2 SD of the controls based on the reported dataset of IgG titers to *Pg*FDC381 and *Pg*SU63 among 10 control individuals [20]. We divided COPD patients into two groups: one is patients whose antibody titers were higher than mean +2SD (High-IgG titer group), and the other includes those whose titers were lower than mean +2SD (Normal-IgG titer group).

Serum C-reactive protein (CRP) and γ-globulin were measured using the High Sensitivity CRP assay (Behring Diagnostics, Westwood, MA, USA), and cellulose acetate electrophoresis, respectively. We assayed 27 cytokines (PDGF, IL-1b, IL-1Ra, IL-2, IL-4, IL-5, IL-6, IL-7, IL-8, IL-9,IL-10, IL-12, IL-13, IL-15, IL-17, eotaxin, FGF, G-CSF, GM-CSF, IFN-γ, IP-10,MCP-1, MIP-1α, MIP-1β, RANTES, TNF-α and VEGF) in 62 patients using a multiplex bead-based immunoassay kit (Bioplex, Bio-Rad Laboratories, Life Science Research Group, Hercules, CA, USA).

### Statistical Analysis

Data were statistically analyzed using the Mann-Whitney U-test and the χ^2^ test with JMP 8.0 software (SAS Campus Drive, Cary, NC, USA) and all results are presented as medians and 25th-75th percentiles. Independent predictors of frequent exacerbations (defined as at least two exacerbations per year) were detected using multivariate logistic regression analysis. All *p-*values are two-sided and *p*<0.05 was considered significant.

## Results

### Baseline Characteristics of Study Patients

Table 1 shows the baseline characteristics of the 93 patients and the prevalence of elevated IgG antibody titer against *P. gingivalis*. The distribution of standardized IgG values were 1.29 (−0.05∼3.75) for PgFDC 381 and 0.06 (−0.47∼0.76) for Pg SU63. The rates of high IgG titer against *Pg*FDC381 and *Pg*Su63 were 52.7% and 23.7%, respectively. Table 2 shows that age, smoking status, smoking index, body mass index, forced expiratory volume in 1 second (FEV_1_), Global Initiative for Chronic Obstructive Lung Disease (GOLD) stage, and MRC did not differ between High-IgG titer group and Normal-IgG titer group. Comorbidities assessed by Charlson Comrbidity index [21] were similar between these two groups. The frequency of using inhaled corticosteroid (ICS) was lower and serum γ-globulin levels were higher in High-IgG titer group than in Normal-IgG titer group, whereas the frequency of using tiotropium and long-acting β2 agonists (LABA), and of serum levels of CRP did not differ (Table 3). None of the patients received salmeterol/fluticasone propionate combination therapy.

**Table 1:** Patients’ baseline characteristics (n = 93).

Age (y)	73 (45–88)
Body mass index (kg/m^2^)	21.7 (14.3–30.5)
Smoking index (pack-years)	57 (20–220)
FEV_1_ (% predicted)	55.0 (19.8–107.1)
GOLD stage, I/II/III/IV	12/46/30/5
High-IgG titer, n (%)	
* Pg*FDC381	49 (52.7)
* Pg*Su63	22 (23.7)

FEV_1_, forced expiratory volume in one second;

GOLD, Global Initiative for Chronic Obstructive Lung Disease;

“High-IgG titer” includes subjects whose titers against *Porphyromonas gingivalis* (*Pg*FDC381 and *Pg*Su63) are above mean+2SD of healthy subsets [20].

Pg, *Porphyromonas gingivalis.* Data are expressed as median (25th–75th percentiles).

**Table 2:** Comparison of baseline characteristics between patients with normal and higher IgG titer against *Porphyromonas gingivalis.*

	Normal-IgG titer(n = 44)	High-IgG titer(n = 49)	*p* value
Age (y)	74 (65–78)	73 (66–78)	0.83
Smoking status (Former : Current)	37∶7	43∶6	0.77
Smoking index (pack-years)	60 (47–102)	53 (40–80)	0.07
Body mass index (kg/m^2^)	21.3 (19.5–23.3)	22.0 (20.1–23.5)	0.46
FEV_1_ (% predicted)	54.4 (39.5–66.7)	56.5 (42.4–72.4)	0.39
GOLD stage (I/II/III/IV)	5/23/13/3	7/23/17/2	0.94
Numbers of patients with frequent exacerbations in theprevious year, n (%)	22 (50.0)	24 (49.0)	1.00
MRC	1 (1–2)	1 (1–2)	0.86
Charlson comorbidity index	2 (1–3)	2 (1–3)	0.76

High-IgG titer group includes subjects whose titers against *Porphyromonas gingivalis* (*Pg*FDC381 and/or *Pg*Su63) are above mean+2SD of healthy subsets [20].

“Frequent exacerbations” are defined as ≥2 exacerbations per year.

FEV_1_, forced expiratory volume in one second; GOLD, Global Initiative for Chronic Obstructive Lung Disease.; MRC, British Medical Research Council. Data are expressed as medians (25th–75th percentiles).

**Table 3:** Inflammatory markers in blood and use of COPD medication in patients between patients with normal and higher IgG titer against *Porphyromonas gingivalis.*

	Normal-IgG titer(n = 44)	High-IgG titer(n = 49)	*p* value
Blood			
γ-globulin, g/dL	1.04 (0.94–1.18)	1.20 (1.05–1.42)	0.0007
C-reactive protein, mg/L	0.82 (0.30–1.86)	0.94 0.36–1.71)	0.66
Use of COPD medications, n(%)			
Inhaled corticosteroid	23 (52.3)	15 (30.6)	0.038
Tiotropium	20 (45.5)	17 (34.7)	0.40
Long-acting β2 agonist	15 (34.1)	18 (36.7)	0.83
Salmeterol/fluticasone combination	0 (0)	0 (0)	1.00

High-IgG titer group includes subjects whose titers against *Porphyromonas gingivalis* (*Pg*FDC381 and/or *Pg*Su63) are above mean+2SD of healthy subsets [20].

Data area expressed as medians (25th–75th percentiles).

### Relationship between Elevated IgG Antibody Titer against *P. gingivalis* and Exacerbation Frequency

**Figure 1:**
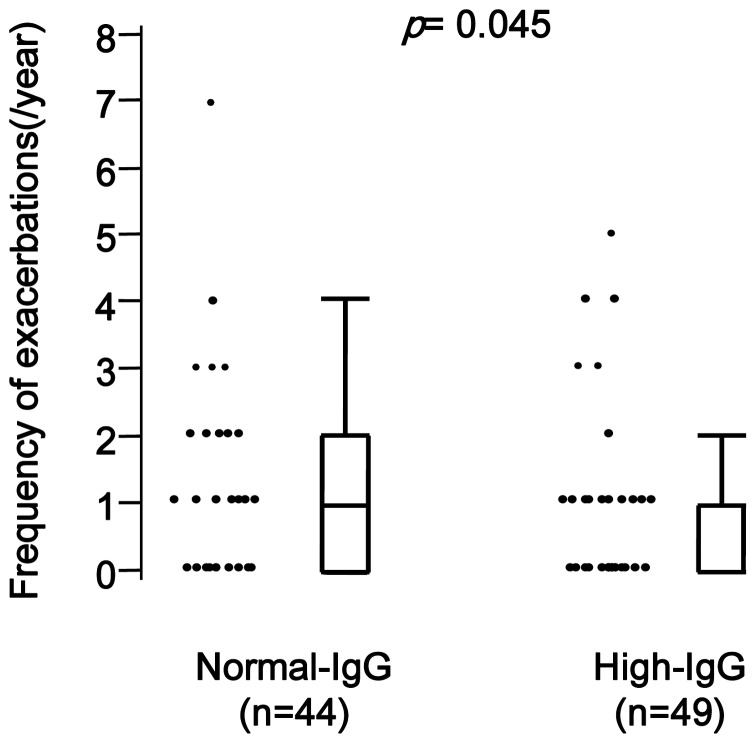
Frequency of exacerbations of patients with COPD and elevated serum IgG antibody titer against *Porphyromonas gingivalis*. Annual frequency of exacerbation is lower in patients with higher IgG titer than with normal IgG titer (1.2 vs. 0.8/year, p  = 0.045).

We hypothesized that more periodontitis lead to more exacerbations, but in contrast to our hypothesis, exacerbations were less frequent in High-IgG titer group than in Normal-IgG titer group (0.8 vs. 1.2 per year, *p*  = 0.045, Figure 1 and Table 4). Moreover, the rate of patients who experienced frequent exacerbations (at least two per year) was also lower in High-IgG titer group than in Normal-IgG titer group (14.3% vs. 38.6%, *p*  = 0.009, Table 4).

**Table 4:** Frequency of exacerbations and elevated serum IgG antibody titer against *Porphyromonas gingivalis.*

	Normal-IgG titer(n = 44)	High-IgG titer(n = 49)	*p* value
Exacerbation frequency, per year			
Median (25^th^–75th percentiles)	1 (0–2)	0 (0–1)	0.045
Mean	1.2	0.8	
Rate of patients with frequent exacerbations, n (%)	17 (38.6)	7 (14.3)	0.009

High-IgG titer group includes subjects whose titers against *Porphyromonas gingivalis* (*Pg*FDC381 and/or *Pg*Su63) are above mean+2SD of healthy subsets [20].

Frequent exacerbations” are defined as ≥2 exacerbations per year.

### Multivariate Analysis to Detect Independent Predictors of Frequent Exacerbation

We performed multivariate logistic regression analysis to determine whether being normal IgG antibody titer against *P. gingivalis* could be an independent risk factor for frequent exacerbations (Table 5). This analysis included the occurrence of frequent exacerbations as a dependent categorical variable and higher IgG antibody titer against *P. gingivalis*, age, smoking status, body mass index, γ-globulin, CRP, use of ICS, tiotropium and LABA, FEV_1_(%predicted) and a history of exacerbations in the year before entry as independent variables. We found that a history of exacerbation in the previous year and normal IgG antibody titer against *P. gingivalis* significantly increased the occurrence of exacerbations (Relative Risk [RR] 4.43, 95% confidence interval [95%CI] 1.20–19.6, *p*  = 0.025; RR 5.27, 95%CI 1.30–25.7, *p  = *0.019, respectively).

**Table 5:** Multivariable logistic regression analysis to identify risk factors for frequent exacerbation.

Variables	RR	95%CI	*p*-value
Age, per increase of 1 year	1.00	0.93–1.09	0.90
Smoking index, per increase of 1 pack-year	0.98	0.96–1.00	0.10
Body mass index, per increase of 1 kg/m^2^	0.89	0.70–1.12	0.34
CRP, per increase of 1 µg/ml	0.99	0.99–1.00	0.70
γ-globulin, per increase of 1 g/dL	2.07	0.17–25.1	0.56
ICS, yes/no	2.01	0.57–7.55	0.28
Tiotropium, yes/no	1.53	0.41–5.80	0.52
LABA, yes/no	1.31	0.33–5.20	0.69
%FEV_1_, per increase of 1%	0.99	0.94–1.02	0.49
Exacerbations in previous year, yes/no	4.43	1.20–19.6	0.025
Normal IgG antibody titer against*Porphyromonas gingivalis.*, yes/no	5.27	1.30–25.7	0.019

Frequent exacerbation is defined as ≥2 exacerbations per year.

RR, relative risk; CI, confidence interval; CRP, C-reactive protein; ICS, inhaled corticosteroid; LABA, long-acting β2 agonist.

### Relationships between Elevated IgG Antibody Titer against *P. gingivalis* and Serum Inflammatory Cytokines: Subanalysis of Immune Status among 62 Patients

Tables S1 and S2 show the results of a comparison of 27 cytokines in 62 patients placed in groups that were higher-IgG titer or normal-IgG titer against *P. gingivalis* according to the multiplex bead-based immunoassay. Levels of serum IL-4 and IL-7 were significantly higher in Normal-IgG titer group than in High-IgG titer group, whereas the other cytokines did not significantly differ between them.

## Discussion

We demonstrated that the frequency of exacerbations was higher among patients with COPD whose antibody titer against *P. gingivalis* was normal compared with those whose antibody titer against *P. gingivalis* was higher. Multivariate logistic regression analysis showed that being normal-IgG titer against *P. gingivalis* was independently associated with frequent exacerbations during the following year after adjustment for established exacerbation-related factors such as a history of exacerbation [2], FEV_1_ [22], [23] and the use of COPD medications [24], [25]. These findings contradicted our hypothesis that periodontitis is a risk factor for COPD exacerbation. Nevertheless, the present findings are important because, to our knowledge, this is the first study to demonstrate a relationship between antibodies and COPD exacerbation, and to indicate the value of *P. gingivalis*-related antibodies as a predictor of exacerbation. There were no significant differences between High-IgG titer group and Normal-IgG titer group in age, FEV_1_, GOLD stage, and exacerbation frequencies at the baseline characteristics.

It has been shown that a history of COPD exacerbations is a good predictor for the future exacerbation [2] and our result suggests that the possible mechanism for the frequent exacerbation, thus it seems apparently inconsistent that the frequencies of COPD exacerbation in the previous year are similar in the two groups in the baseline characteristics. However, it is also reported that considerable part of patients who experienced exacerbation in the previous year did not experience COPD exacerbation in the following year [2]. Moreover, we could not specify the timing that the antibody titers increased or decreased. It is possible that the severity of periodontitis, antibody titers or immune status might be different in the previous year and the actual prospective observational period. Further investigation is needed to verify these speculations.

Periodontitis is a disease that afflicts dentate people, so we checked only in cases with teeth. In the dentate patients group, elevated serum IgG antibody titer didn’t associated with exacerbation frequency directly, but frequent exacerbations (at least two per year) was lower in High-IgG titer group than in Normal-IgG titer group (15.6% vs. 44.0%, *p*  = 0.018, Table S3).

Because periodontitis is often complicated with diabetic mellitus [26], [27] or other systemic diseases [16], [17], [28]–[30], we also checked the comorbidities by using Charlson Comorbidities index. There was no difference between High-IgG titer group and Normal-IgG titer group. In this study population, comorbidities of COPD were not influenced on the periodontitis.

The importance of predictors of exacerbation is becoming recognized because identifying patients with susceptibility to frequent exacerbations allows the design of an aggressive prevention strategy [2]. We previously showed that an impaired swallowing reflex can predict frequent exacerbations [7]. Assuming that the aspiration of bacteria into the lungs especially among patients with an impaired swallowing reflex can cause lower respiratory tract infection, we postulated that the common oral infection, periodontitis, is associated with exacerbation.

Although a diagnosis of periodontitis requires a systematic approach including a comprehensive periodontal examination by a dentist, we used IgG antibody titers against periodontal pathogens as a surrogate marker. The validity of antibodies related to periodontitis in the management of periodontitis has been confirmed [15], [20]. Serum pathogen-specific antibody levels reflect amounts of periodontal bacteria, and they are useful to assess the effects of periodontitis treatment [31], [32].

Because periodontitis frequently occurs in patients with COPD [13], we assumed that such patients could be chronically exposed to periodontitis-related pathogens. Furthermore, *P. gingivalis* is the most frequently found pathogen in periodontitis [15]. Thus, serum levels of *P. gingivalis*-related antibody might reflect the ability to generate specific immune responses against infection with bacterial pathogens. This could be supported by the finding that serum γ-globulin levels were higher in High-IgG titer group than in Normal-IgG titer group. As bacterial infection is a major cause of COPD exacerbations [33], we speculated that an insufficient humoral immune response against bacteria in patients whose antibody titer against *P. gingivalis* was normal would be related to an increase in exacerbation frequency.

Our subanalysis of 27 cytokines using the multiplex bead-based immunoassay in 62 patients with COPD found higher serum levels of IL-4 and IL-7 in Normal-IgG titer group than High-IgG titer group (Table S1 and S2). Serum IL-4 level was measured by multiple testing in this study. The differences in the cytokine concentrations might be too small to attach a biological significance. However, some studies suggested that slight difference in the IL-4 concentration exerted biological difference [34], [35]. We should be prudent to interpret these results.

The fact that higher serum levels of IL-4 and IL-7 in Normal-IgG titer group than High-IgG titer group seems to be contradictory to the fact that IL-4 functions as a B cell growth factor [36] and IL-7 simulates the proliferation of lymphoid progenitors [37]. However, we speculated that lower IgG could be a result from insufficient IgG production in spite of the intense producing stimuli such as IL-4 and IL-7 in some way that we could not identify in this research, and impaired negative feedback system on cytokine production might cause the higher level of IL-4 and IL-7. Another possible explanation is that Th2 cytokine suppression might influence our results. Papi A et al reported that airway eosinophil counts were increased during COPD exacerbations associated with virus detection [38] and some studies showed that virus infection lead to increase of IL-4 and made Th1/2 balance skew toward Th2 cytokines [39]. As a whole, it is possible that higher IgG titer and lower IL-4 might lead to systemic suppression of Th2 immune programming resulting in the fewer exacerbation frequencies. However, the differences in these cytokines in this study were not so prominent and it is difficult to conclude definitely. Further studies are needed to clarify these speculations.

We previously showed that abnormal swallowing reflexes are associated with frequent exacerbations [7]. In the present study, we examined swallowing reflexes in 56 patients and separately evaluated the relationship between *P. gingivalis*-related antibody titers and exacerbation frequency in groups with normal and impaired swallowing reflexes. Table S4 shows that being normal-IgG titers for *P. gingivalis*-related antibody tended to increase exacerbation frequency in both groups. Although drawing a conclusive interpretation from the small sample cohort in the present study is difficult, we believe that the absence of *P. gingivalis*-related antibody contributes to exacerbation frequency regardless of the status of the swallowing reflex.

To evaluate the relationship between *P. gingivalis*-related antibody titers and airway inflammation, we induced sputum in 46 patients by having them inhale 3% hypertonic saline using an ultrasonic nebuliser (MU-32, Azwell Inc., Osaka, Japan). Table S5 shows that the total cell counts, profiles of inflammatory cells and inflammatory cytokines (IL-8 and TNF-α) in the supernatants did not differ between Normal-IgG titer group and High-IgG titer group. This finding supports the notion that antibody positivity reflects chronic low-level exposure to periodontitis-related pathogens rather than acute exaggerated airway inflammation induced by periodontitis-related pathogens.

This study has some limitations. Firstly, we could not find the significant correlation between periodontitis-related antibody titers and exacerbation frequency. (p = 0.1128, Figure S1). We speculated that the antibody titers might be determined by both the severity of the infection and the host reactivity, and COPD exacerbations were affected by various factors such as host defense mechanisms and the intensity of external insults, resulting in the difficulty for detecting the association between antibody titer and exacerbation frequency.

Secondly, since a dentist did not perform a comprehensive periodontal examination, we could not precisely diagnose periodontitis. However, the validity of the periodontitis-related antibody in the management of periodontitis has been confirmed [15], [20]. We thus believe that patients with *Pg* FDC381 or *Pg* SU63 antibody had periodontitis, and that periodontitis itself might not be associated with exacerbation occurrence.

Thirdly, since oral pathogens were not quantitatively cultured, the extent of exposure to periodontal pathogens could not be determined. For example, *peptostreptococcus spp.* and *bacteroides spp.* colonized in the oral cavity and causes airway infection. In addition to that, other bacteria that can colonize in the oral cavity or nasopharynx except for periodontal pathogens such as *Haemophilus inﬂuenzae*, *Moraxella catarrhalis*, *Streptococcus pneumoniae*, *Staphylococcus aureus*, or *Pseudomonas aeruginosa* and Para *Haemophilus inﬂuenzae* were reported to be cultured from sputum at stable state COPD patients [40]. Also, Bafadhel et al. revealed that 55% of exacerbations were associated with bacterial infections and sputum cultures at exacerbations were associated with such bacteria [41], [42]. But their association was still limited and periodontal pathogens were not investigated, so further investigations are needed.

Fourthly, we measured *P. gingivalis*-related antibody only once during an exacerbation-free state. Unlike paired examinations of the antibody titers, we could not discriminate bacterial colonization from new acquisition of pathogens. However, infection with *Pg* FDC381 and *Pg* SU63, both of which are common pathogens in periodontitis [15], mainly results in chronic inflammation of the periodontium. We thus speculated that levels of *P. gingivalis*-related antibody reflect the immune response to chronic infection with this pathogen.

Fifthly, we did not directly investigate the immunological status of the hosts, such as the ability to produce antibody, because a methodology has not yet been established.

### Conclusion

In conclusion, normal-IgG titer for *P. gingivalis*-related antibody can be an independent predictor of exacerbation frequency. Measuring *P. gingivalis*-related antibody titers might be useful to identify patients with susceptibility to frequent exacerbations so that an aggressive strategy can be designed to prevent exacerbation.

## Supporting Information

Figure S1 **Frequency of exacerbations of patients with COPD and serum IgG antibody titer against** ***Porphyromonas gingivalis*** **.** Annual frequency of exacerbation is not correlated with *Porphyromonas gingivalis*-related antibody titers (p = 0.1128).
(TIF)

Table S1 Patients’ baseline characteristics (n  = 62).
(DOC)

Table S2 Comparison of 27 cytokines between patients with normal and higher IgG titer against *Porphyromonas gingivalis*: subanalysis of 62 patients.
(DOC)

Table S3 Frequency of exacerbations and elevated serum IgG antibody titer against *Porphyromonas gingivalis* in dentate patients group.
(DOC)

Table S4 Frequency of exacerbations and elevated serum IgG antibody titer against *Porphyromonas gingivalis* in patients with normal and abnormal swallowing reflexes: subanalysis of 56 patients.
(DOC)

Table S5 Comparison of inflammatory markers in sputum from patients with normal and higher IgG titer against *Porphyromonas gingivalis*: subanalysis of 46 patients.
(DOC)
