# Supplementary figures and images for: Relationship between Periodontitis-Related Antibody and Frequent Exacerbations in Chronic Obstructive Pulmonary Disease
Source: PLoS One. 2012 Jul 11;7(7):e40570. doi: 10.1371/journal.pone.0040570 (PMC3394734; doi:10.1371/journal.pone.0040570)

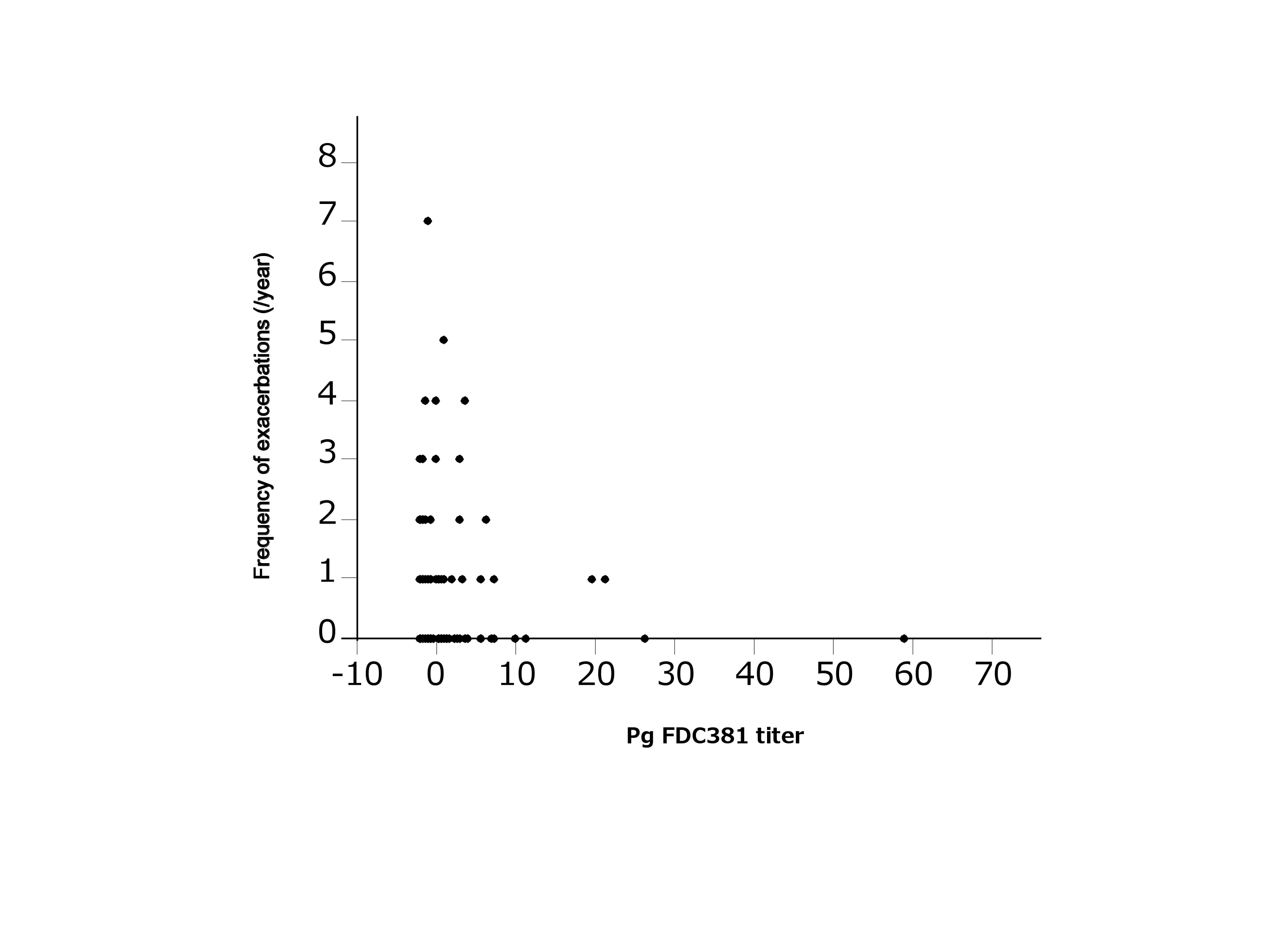

Supplement: Figure S1 — Frequency of exacerbations of patients with COPD and serum IgG antibody titer against Porphyromonas gingivalis . Annual frequency of exacerbation is not correlated with Porphyromonas gingivalis-related antibody titers (p = 0.1128). (TIF) [file pone.0040570.s001.tif]
