# Supplementary material for: Relationship between Periodontitis-Related Antibody and Frequent Exacerbations in Chronic Obstructive Pulmonary Disease
Source: PLoS One. 2012 Jul 11;7(7):e40570. doi: 10.1371/journal.pone.0040570 (PMC3394734; doi:10.1371/journal.pone.0040570)
Supplement: Table S1 — Patients’ baseline characteristics (n = 62). (DOC) [file pone.0040570.s002.doc]

**Table S1. Patients’ baseline characteristics (n = 62).**

| Age (y) | 72 (66-77) |
| --- | --- |
| Body mass index (kg/m^2^) | 21.4 (19.5-23.1) |
| Smoking index (pack-years) | 60 (49-86) |
| FEV_1_ (% predicted) | 54.8 (40.0-66.2) |
| GOLD stage, I/II/III/IV | 7/29/23/3 |
| High-IgG titer, n (%) |  |
| *Pg*FDC381 | 32 (51.6) |
| *Pg*Su63 | 13 (21.0) |

FEV_1_, forced expiratory volume in one second;

GOLD, Global Initiative for Chronic Obstructive Lung Disease;

“High-IgG titer” includes subjects whose titers against *Porphyromonas gingivalis* (*Pg*FDC381 and *Pg*Su63) are above mean+2SD of healthy subsets [20].

Pg, *Porphyromonas gingivalis.* Data are expressed as median (25th-75th percentiles).
