# Supplementary material for: Relationship between Periodontitis-Related Antibody and Frequent Exacerbations in Chronic Obstructive Pulmonary Disease
Source: PLoS One. 2012 Jul 11;7(7):e40570. doi: 10.1371/journal.pone.0040570 (PMC3394734; doi:10.1371/journal.pone.0040570)
Supplement: Table S2 — Comparison of 27 cytokines between patients with normal and higher IgG titer against Porphyromonas gingivalis: subanalysis of 62 patients. (DOC) [file pone.0040570.s003.doc]

**Table S2. Comparison of 27 cytokines between patients with normal and higher IgG titer against *Porphyromonas gingivalis*: subanalysis of 62 patients**.

| Cytokine (pg/mL) | Normal-IgG titer (n =30) | High-IgG titer (n =32) | *p* value |
| --- | --- | --- | --- |
| Eotaxin | 153.7 (121.9-191.0) | 169.9 (114.0-202.3) | 0.47 |
| bFGF | N.D | N.D | - |
| G-CSF | 2.22 (1.79-3.24) | 2.41 (1.88-3.17) | 0.84 |
| GM-CSF | 18.2 (11.1-26.5) | 16.3 (10.5-22.2) | 0.41 |
| IFN-γ | 30.9 (25.9-41.9) | 30.9 (27.5-35.5) | 0.90 |
| IL-1b | 0.94 (0.78-1.2) | 0.91 (0.74-1.0) | 0.42 |
| IL1-Rα | 37.3 (29.6-46.3) | 34.9 (25.1-44.7) | 0.41 |
| IL-2 | N.D | N.D | - |
| IL-4 | 1.12 (1.02-1.24) | 0.98 (0.9-1.22) | 0.039 |
| IL-5 | 1.2 (0.95-1.67) | 1.27 (0.97-1.58) | 0.87 |
| IL-6 | 3.24 (2.61-4.53) | 3.41 (2.50-4.28) | 0.90 |
| IL-7 | 5.70 (3.63-6.51) | 4.27 (3.63-6.17) | 0.0311 |
| IL-8 | 8.64 (7.3-9.85) | 8.69 (6.40-10.2) | 0.67 |
| IL-9 | 6.71 (5.59-12.9) | 6.24 (3.41-18.4) | 0.56 |
| IL-10 | 0.12 (0-0.59) | 0.19 (0.02-0.29) | 0.67 |
| IL-12 | 6.77 (3.74-10.7) | 6.49 (4.30-8.47) | 0.96 |
| IL-13 | 4.0 (3.04-5.68) | 3.9 (2.90-4.73) | 0.55 |
| IL-15 | N.D | N.D | - |
| IL-17 | 1.36 (0-2.56) | 0.86 (0-3.67) | 0.92 |
| IP-10 | 496.8 (412.6-578.3) | 487.4 (352.5-675.3) | 0.84 |
| MCP-1 | 13.9 (10.4-22.7) | 13.9 (10.3-25.0) | 0.91 |
| MIP-1a | N.D | N.D | - |
| MIP-1b | 103.3 (82.2-128.6) | 95.0 (69.8-144.8) | 0.83 |
| PDGF* | 6.6 (4.3-9.4) | 5.9 (4.1-8.7) | 0.49 |
| RANTES | 1.8 (1.4-2.3) | 1.6 (1.4-2.1) | 0.58 |
| TNF-α | N.D | N.D | - |
| VEGF | 93.8 (49.6-128.5) | 78.9 (58.9-116.6) | 0.76 |

*Cytokine values shown pg/mL except for PDGF (ng/mL). High-IgG titer group includes subjects whose titers against *Porphyromonas gingivalis* (*Pg*FDC381 and/or *Pg*Su63) are above mean+2SD of healthy subsets [20].

bFGF, basic fibroblast growth factor; G-CSF, granulocyte-colony stimulating factor; GM-CSF, granulocyte-macrophage-colony stimulating factor; IFN-γ, interferon-γ; IL, interleukin; IL-1Rα, interleukin1 receptor α ; IP-10, interferon-inducible protein 10; MCP-1, monocyte chemotactic protein-1; MIP, macrophage inflammatory protein; PDGF, platelet-derived growth factor; RANTES, Regulated upon activation, normal T-cell expressed, and secreted; TNF, tumor necrotic factor; VEGF, vascular endothelial growth factor; N.D., not detected. Data are expressed as medians (25th-75th percentile)
