# Supplementary material for: Relationship between Periodontitis-Related Antibody and Frequent Exacerbations in Chronic Obstructive Pulmonary Disease
Source: PLoS One. 2012 Jul 11;7(7):e40570. doi: 10.1371/journal.pone.0040570 (PMC3394734; doi:10.1371/journal.pone.0040570)
Supplement: Table S3 — Frequency of exacerbations and elevated serum IgG antibody titer against Porphyromonas gingivalis in dentate patients group. (DOC) [file pone.0040570.s004.doc]

**Table S3. Frequency of exacerbations and elevated serum IgG antibody titer against *Porphyromonas gingivalis* in dentate patients group**.

|  | | Normal-IgG titer (n= 25) | High-IgG titer (n = 32) | *p* value |
| --- | --- | --- | --- | --- |
| Exacerbation frequency, per year | |  |  |  |
|  | Median (25th-75th percentiles) | 1 (0-2) | 0 (0-1) | 0.104 |
|  | Mean | 1.4 | 0.8 |  |
| Rate of patients with frequent exacerbations, n (%) | | 11 (44.0) | 5 (15.6) | 0.018 |

High-IgG titer group includes subjects whose titers against *Porphyromonas gingivalis* (*Pg*FDC381 and/or *Pg*Su63) are above mean+2SD of healthy subsets [20].

“Frequent exacerbations” are defined as ≥2 exacerbations per year.
