# Supplementary material for: Relationship between Periodontitis-Related Antibody and Frequent Exacerbations in Chronic Obstructive Pulmonary Disease
Source: PLoS One. 2012 Jul 11;7(7):e40570. doi: 10.1371/journal.pone.0040570 (PMC3394734; doi:10.1371/journal.pone.0040570)
Supplement: Table S4 — Frequency of exacerbations and elevated serum IgG antibody titer against Porphyromonas gingivalis in patients with normal and abnormal swallowing reflexes: subanalysis of 56 patients. (DOC) [file pone.0040570.s005.doc]

**Table S4. Frequency of exacerbations and elevated serum IgG antibody titer against *Porphyromonas gingivalis* in patients with normal and abnormal swallowing reflexes: subanalysis of 56 patients.**

| **Patients with normal swallowing reflex (n = 37)** | | |  |  |
| --- | --- | --- | --- | --- |
|  | | Normal-IgG titer (n = 15) | High-IgG titer (n = 22) | *p* value |
| Exacerbation frequency, per year | |  |  |  |
|  | Median (25th-75th percentile) | 1 (0-2) | 0.5(0-1) | 0.13 |
|  | Mean | 1.2 | 0.7 |  |
| Rate of patients with frequent exacerbations, n (%) | | 6 (40.0) | 3 (13.6) | 0.12 |
| **Patients with abnormal swallowing reflex (n = 19)** | | |  |  |
|  | | Normal-IgG titer (n = 10) | High-IgG titer (n = 9) | *p* value |
| Exacerbation frequency, per year | |  |  |  |
|  | Median (25th-75th percentile) | 2 (0.8-2.3) | 1 (0-1.5) | 0.22 |
|  | Mean | 1.7 | 1.1 |  |
| Rate of patients with frequent exacerbations, n (%) | | 6 (60.0) | 2 (22.2) | 0.10 |

High-IgG titer group includes subjects whose titers against *Porphyromonas gingivalis* (*Pg*FDC381 and/or *Pg*Su63) are above mean+2SD of healthy subsets [20].

“Frequent exacerbations” are defined as ≥ 2 exacerbations per year.
