# Supplementary material for: Relationship between Periodontitis-Related Antibody and Frequent Exacerbations in Chronic Obstructive Pulmonary Disease
Source: PLoS One. 2012 Jul 11;7(7):e40570. doi: 10.1371/journal.pone.0040570 (PMC3394734; doi:10.1371/journal.pone.0040570)
Supplement: Table S5 — Comparison of inflammatory markers in sputum from patients with normal and higher IgG titer against Porphyromonas gingivalis: subanalysis of 46 patients. (DOC) [file pone.0040570.s006.doc]

**Table S5. Comparison of inflammatory markers in sputum from patients with normal and higher IgG titer against *Porphyromonas gingivalis*: subanalysis of 46 patients.**

|  | | | Normal-IgG titer (n = 27) | High-IgG titer (n = 19) | *p* value |
| --- | --- | --- | --- | --- | --- |
| Inflammatory cell profile | |  | |  |  |
|  | Total cells (×10^5^/mL) | 15.8 (9.86-22.3) | | 19.1 (12.4-28.7) | 0.33 |
|  | Neutrophils (×10^5^/mL) | 10.1 (7.18-16.3) | | 10.8 (4.52-21.6) | 0.95 |
|  | Monocytes (×10^5^/mL) | 3.98 (2.44-8.42) | | 3.52 (1.62-6.45) | 0.60 |
| IL-8 (pg/mL) | | 17.6 (5.85-43.8) | | 19.5 (8.24-83.6) | 0.61 |
| TNF-α (pg/mL) | | 13.7 (10.2-99.9) | | 21.5 (11.8-98.7) | 0.48 |

High-IgG titer group includes subjects whose titers against *Porphyromonas gingivalis* (*Pg*FDC381 and/or *Pg*Su63) are above mean+2SD of healthy subsets [20].

IL-8, interleukin-8; TNF-α, tumor necrotic factor alpha.

Data area expressed as medians (25th-75th percentiles).
